# The Grasping Side of Odours

**DOI:** 10.1371/journal.pone.0001795

**Published:** 2008-03-19

**Authors:** Federico Tubaldi, Caterina Ansuini, Roberto Tirindelli, Umberto Castiello

**Affiliations:** 1 Department of General Psychology, University of Padua, Padua, Italy; 2 Department of Neuroscience, University of Parma, Parma, Italy; 3 Department of Psychology, Royal Holloway, University of London, Egham, United Kingdom; Lund University, Sweden

## Abstract

**Background:**

Research on multisensory integration during natural tasks such as reach-to-grasp is still in its infancy. Crossmodal links between vision, proprioception and audition have been identified, but how olfaction contributes to plan and control reach-to-grasp movements has not been decisively shown. We used kinematics to explicitly test the influence of olfactory stimuli on reach-to-grasp movements.

**Methodology/Principal Findings:**

Subjects were requested to reach towards and grasp a small or a large visual target (i.e., precision grip, involving the opposition of index finger and thumb for a small size target and a power grip, involving the flexion of all digits around the object for a large target) in the absence or in the presence of an odour evoking either a small or a large object that if grasped would require a precision grip and a whole hand grasp, respectively. When the type of grasp evoked by the odour did not coincide with that for the visual target, interference effects were evident on the kinematics of hand shaping and the level of synergies amongst fingers decreased. When the visual target and the object evoked by the odour required the same type of grasp, facilitation emerged and the intrinsic relations amongst individual fingers were maintained.

**Conclusions/Significance:**

This study demonstrates that olfactory information contains highly detailed information able to elicit the planning for a reach-to-grasp movement suited to interact with the evoked object. The findings offer a substantial contribution to the current debate about the multisensory nature of the sensorimotor transformations underlying grasping.

## Introduction

Reach and grasp movements are amongst the most common actions we perform in our everyday lives. To perform this kind of action, different sensory modalities are used in concert to perceive and interact with multimodally specified objects and events [1]–[5].

The visual system provides information about object location, size, shape, and orientation, and also about the movement of one's hand towards the object [6]–[8]. The haptic system provides information about object weight and texture [9], confirms target acquisition, modulates grip force for stable grasp [10]–[12], and contributes to detect potential collisions with other objects in the environment. Action-generated sounds and noises are very common in a natural environment and touch related sounds can also provide information about the structure of surfaces [13], [14].

Although the above evidence suggests that the motor system takes into account streams of information encoded in different modalities, it is customary to study sensory systems in isolation. However, most real-life situations require that these sensory systems provide us with integrated cues about object properties and recent antecedents seem to suggest that such integration is particularly relevant when reaching to grasp an object [15]–[19]. For instance, when estimating where a hand is in space, visual and proprioceptive information are available. These two sources of information are integrated in a way that minimizes the uncertainty in the estimate, which in turn is used to plan a goal-directed movement [15]–[17], [20], [21]. Adding sound contact cues on motor performance when reaching to grasp an object facilitates and fine-tunes action performance [18], [19], [21]–[24].

An aspect which has been largely neglected in terms of the multisensory processes underlying reach to-grasp movements concerns chemosensory information. One study in our laboratory considered reach-to-grasp movements performed in the presence of an olfactory task-irrelevant stimulus. The olfactory stimulus could evoke an object of a smaller or larger dimension than the target object. In these circumstances, the maximum distance between the index finger and thumb (i.e., maximum hand aperture) was affected. If the olfactory stimulus evoked an object smaller than the target, then maximum hand aperture was smaller than when no-odour was delivered. If the olfactory stimulus evoked an object larger than the target, then maximum hand aperture was larger than when grasping occurred in the absence of olfactory information [25].

Although suggestive of the potential influence olfactory information may have on reach-to-grasp movements, the dependent measure used in this preliminary observation (i.e., maximum hand aperture) did not allow for a precise examination of three critical aspects. First, it does not permit a full understanding of how detailed the motor commands embedded within the ‘grasp’ plan elicited by the object's olfactory representation are. In this respect, recording detailed kinematics at the level of individual digits may shed more light on this aspect. If the motion of individual fingers is modulated by the olfactory information, then the ‘grasp’ plan elicited by the olfactory representation may consider the structure of the object associated with the odour. Second, maximum hand aperture is a measure which does not allow to ascertain how olfactory interference fully manifests within a complex sensory-motor system such as that sub-serving visual grasping. An index quantifying the intrinsic relations amongst fingers, such as the pattern of hand motion covariation (i.e., the extent to which the motion of digits' single joints is coordinated into synergies [26], [27]), may be needed. If an odour affects the pattern of hand motion covariation, then olfactory-induced destabilization of motion synergies amongst fingers would be a potent index of interference. Conversely, if an odour leaves unchanged the pattern of hand motion covariation, then no inferences about olfactory type of interference could be drawn. Finally, maximum hand aperture is a time-locked kinematic parameter (i.e., occurs at 50–60% of reaching duration when grasping under natural conditions) which does not allow to determine with a high temporal resolution when the olfactory and the visual information integrate en-route for action control. In this respect, by looking at the entire time course of action would allow to determine when the olfactory and the visual information do integrate.

**Figure 1:**
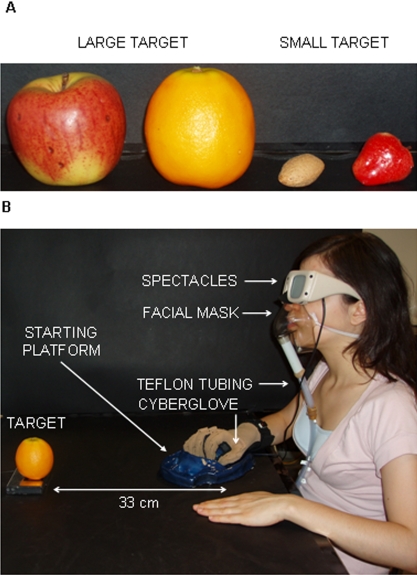
The visual targets and the experimental set up. (A) The visual targets defined as ‘large’ were an apple and an orange, whereas those defined as ‘small’ were an almond and a strawberry. (B) Legends indicate the parts composing the experimental set up.

**Figure 2:**
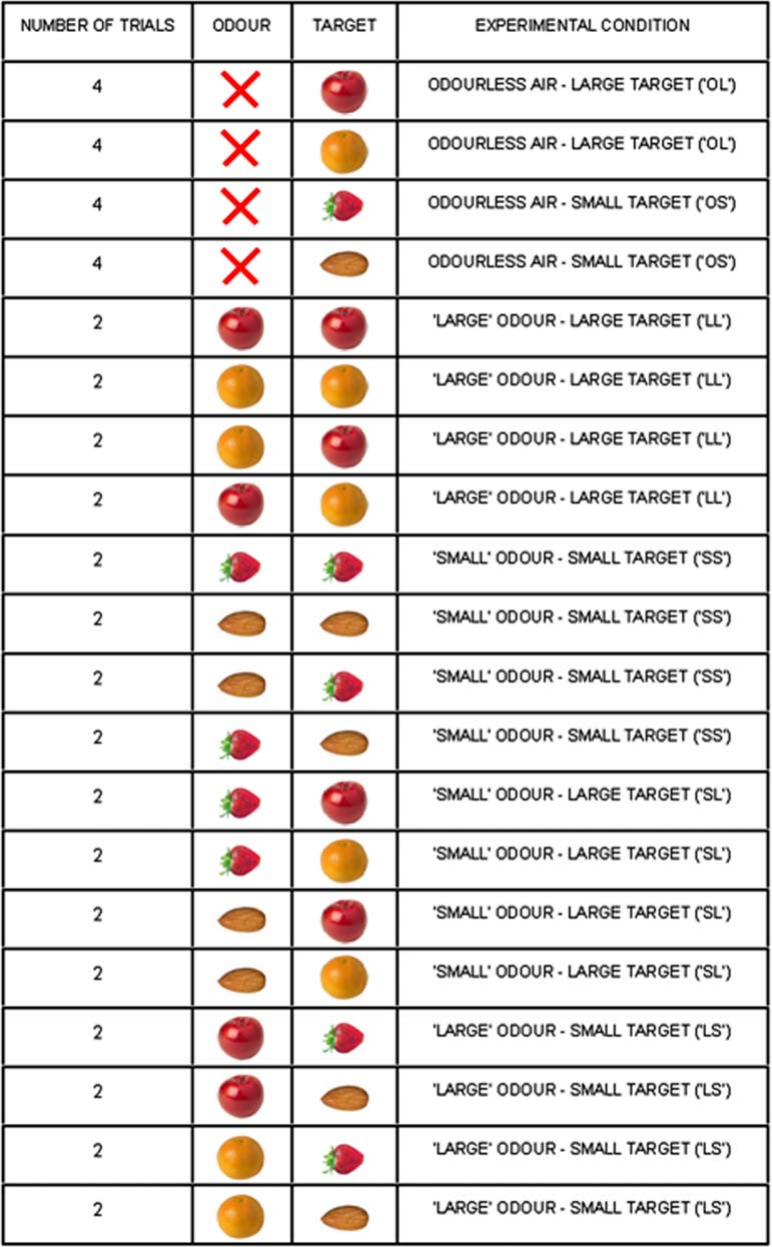
Odour-target combination for each experimental condition. From left to right columns report the number of trials for each odour/target combination, the type of odour, the type of target, and the experimental conditions.

With this in mind, we set out to investigate detailed hand kinematics along the entire time course of a reach-to-grasp movement towards visual targets of different size eliciting different types of grasp (Fig. 1A) in the absence or in the presence of preceding olfactory information. Specifically, we recorded angular excursion at the metacarpal phalangeal (*mcp*) and proximal interphalangeal (*pip*) joints for all five digits, and abduction angles between digits by means of a CyberGlove (Fig. 1B). For the odourless conditions, subjects reached towards and grasped either a small or a large visual target in the absence of preceding olfactory information by using a precision grip and a power grip, respectively. These conditions were termed respectively ‘OS’ and ‘OL’ (Fig. 2). For the congruent conditions, before movement initiation an odour evoking an object that if grasped would require the same type of grasp as the visual target was delivered. These conditions were named ‘SS’ and ‘LL’, respectively (Fig. 2). For the incongruent conditions, before movement initiation an odour evoking an object that if grasped would require a different type of grasp as the visual target was delivered. For the ‘SL’ condition, an odour associated with an object requiring a precise grasp was presented with a visual target requiring a whole hand grasp (Fig. 2). For the ‘LS’ condition, an odour associated with an object requiring a whole hand grasp was presented with a target requiring a precision grip (Fig. 2).

Capitalizing on the effects of olfactory information on reach-to-grasp movements previously reported [25] we hypothesized that an odour delivered before movement initiation might be able to trigger a motor plan reflecting the size of the object associated with the odour (i.e., power grip for a large sized stimulus vs. precision grip for a small sized stimulus). Therefore, we expect that the size information carried by the odour would affect kinematics differently depending on the congruency between the motor plan elicited by the ‘size’ of the delivered odour and that elicited by the size of the visual target. Specifically we foresee that for the incongruent conditions the motor plan dictated by the visual target should interfere with the motor plan elicited by the olfactory stimulus. For instance, if the delivery of a ‘large’ odour is followed by the presentation of a small visual target, then angular values at both fingers' joints and abductions would be greater than when no olfactory information is given. Conversely, we expected that when an odour associated with a small object is delivered and the target is large, angular values would be smaller than when no-odour is administered. For the congruent conditions, in which both the olfactory and visual information elicit a similar motor plan, the pattern of fingers' joints and abductions should be more pronounced than when no olfactory information is provided. Finally, in order to specifically test the extent of the influence olfactory information may have on the unfolding of the reach-to-grasp movements we also evaluated hand motion covariation patterns. The comparison of hand motion covariation for the congruent and the incongruent conditions with the no-odour conditions should give a measure of how the olfactory stimulus influences the degree of coordination amongst digits.

To sum up, the aim of the present study was to address three critical and interrelated questions: (i) whether central mechanisms for the visual guidance of grasping are sensitive to olfactory information; (ii) whether the integration of an olfactory stimulus eliciting a hand conformation similar to that elicited by the visual target facilitates the production of a hand posture tailored for the visual target; and (iii) whether delivering an olfactory stimulus - eliciting a hand conformation different from that called by the visual target - reveals interference mechanisms which are played out on the functional organization of individual finger joints.

## Results

### The Effect of Size on Hand Shaping

**Figure 3:**
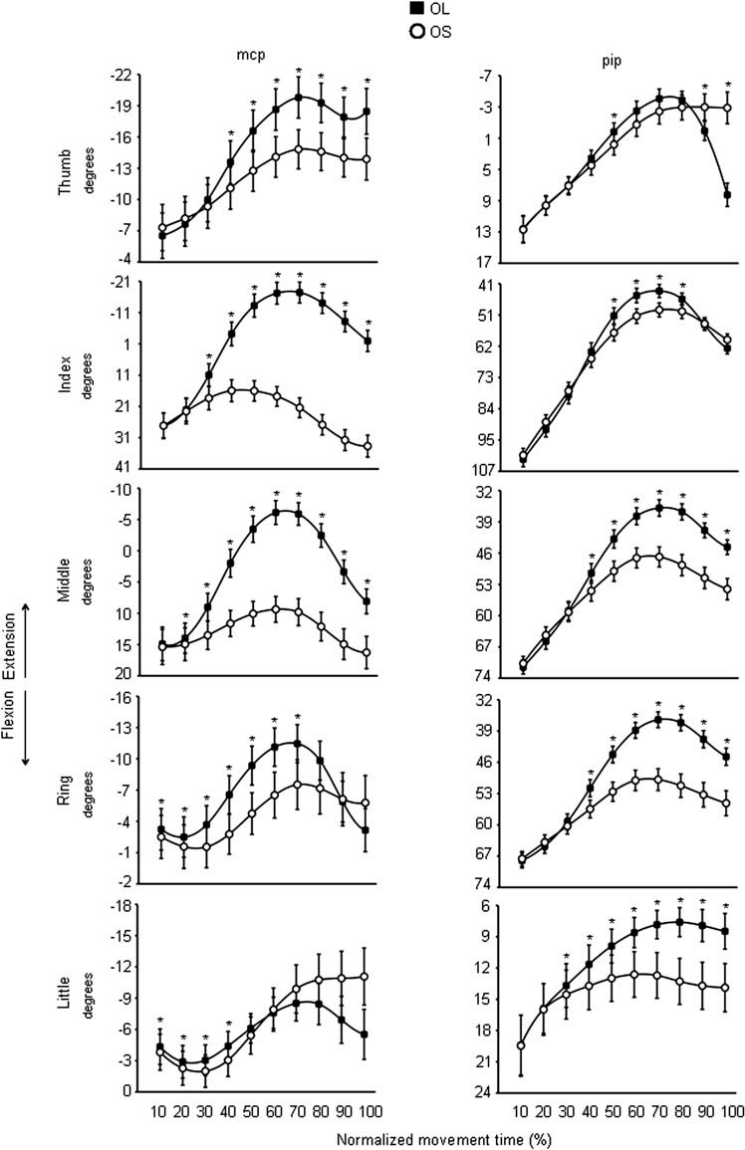
Time course of fingers motion during reaching in the absence of olfactory stimuli. Each trace corresponds to the average angular excursion for the *mcp* (left panels) and *pip* (right panels) joints of the thumb, index, middle, ring, and little fingers for the ‘OL’ (black squares) and the ‘OS’ (white circles) conditions. Bars represent mean standard error. Positive values correspond to finger flexion, whereas negative values correspond to finger extension. Asterisks indicate significant results (*p*<.05) for the comparisons between the ‘OL’ and the ‘OS’ conditions at different epochs of normalized movement time. OL = Odourless air-Large target; OS = Odourless air-Small target.

**Figure 4:**
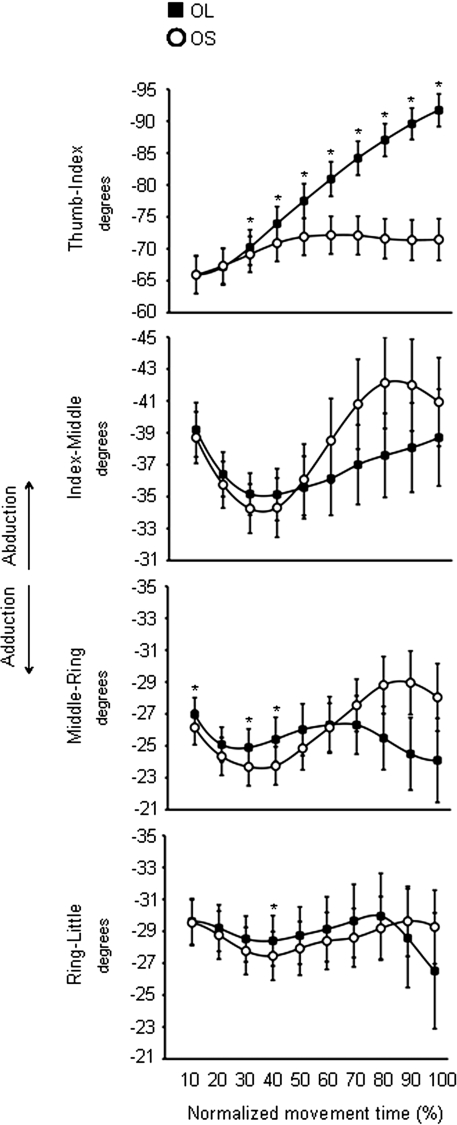
Time course of abduction angle between fingers during reaching in the absence of olfactory stimuli. Each trace corresponds to the average abduction angle for the ‘OL’ (black squares) and the ‘OS’ (white circles) conditions. Bars represent mean standard error. Increase in negative values correspond to bigger abduction (i.e., increase of digits' angular distance). Asterisks indicate significant results (*p<*.05) for the comparisons between the ‘OL’ and the ‘OS’ conditions at different epochs of normalized movement time. OL = Odourless air-Large target; OS = Odourless air-Small target.

Here we present the effects of target size on hand shaping as derived from the conditions in which the visual targets are presented in the absence of preceding olfactory information. This is an important aspect of the present study because in order to ascertain the effects of olfactory information in terms of ‘size’ on hand shaping it is necessary to demonstrate that the size of the visual target does affect hand shaping. In this respect, significantly different kinematic patterns of hand shaping for the small and the large targets were found. As shown in Fig. 3 the *mcp* joint for the thumb was more extended for the large than for the small target from 40% to the end of the movement. The *mcp* joint for the index and the middle fingers was significantly more extended for the large than for the small target throughout the entire movement. For the ring and little fingers no significant differences with respect to target size were found from 70 and from 40% up to the end of movement duration, respectively (Fig. 3). A similar pattern was also evident for the *pip* joints of all fingers (except for the thumb), but differences related to target size became evident at a later time than for the *mcp* joints. The *pip* joint of the thumb was more flexed for the large than for the small target during the last epoch (90–100%). The thumb-index abduction angle was greater for the large than for the small target from 30 up to 100% of movement duration (Fig. 4). Similar size effects were also evident for the middle-ring and the ring-little abduction angles from 10 to 40% of movement duration (Fig. 4). In summary, the fingers were more extended when preparing to grasp a larger than a smaller target whereas the thumb was more flexed for the large than for the small target. This signifies that the size of the visual target was taken into account when planning the motion of all digits.

### The Effect of Odours on Hand Shaping

Here we describe the specific effects of odour ‘size’ on hand shaping. Specifically in the following sections we report on the effects of odour ‘size’ on the digits' angular excursion and abduction angles.

#### Grasping a large target

**Figure 5:**
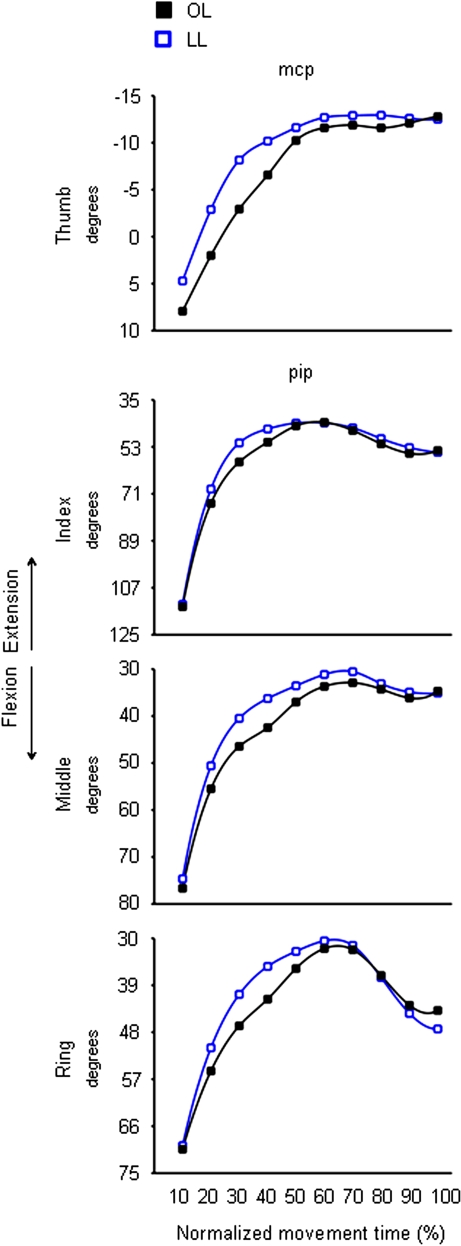
Time course of finger motion during reaching for the large target either in the absence or in the presence of an odour evoking a large object. Each trace corresponds to the average angular excursion of a representative subject (Subject 15) for the *mcp* joint of the thumb and the *pip* joint of the index, middle, and ring fingers when performing the ‘OL’ (black squares) and the ‘LL’ (blue squares) conditions. Positive values correspond to finger flexion whereas negative values correspond to finger extension. OL = Odourless air-Large target; LL = ‘Large’ odour-Large target.

For the congruent ‘LL’ condition, the *pip* joint of the index, middle and ring fingers was more extended than for the ‘OL’ condition (Fig. 5). This effect was particularly evident at the very beginning of movement duration (i.e., at 10–20% for both the index and the ring finger, and at 20% for the middle finger) (Table 1). A similar effect was exhibited by the *mcp* joint of the thumb which was more extended for the ‘LL’ than for the ‘OL’ condition at 20% of movement duration (Fig. 5 and Table 1). For these joints, after 20% of movement duration, no differences when comparing ‘LL’ and the ‘OL’ conditions were evident.

**Table 1:** Average angular excursions at different epochs of normalized movement time.

	normalized movement time (%)			
	10		20	
	ol	ll	ol	ll
joint				
tmcp			−7(2)	−8(2.5)^*^
ipip	103(2.5)	101.5(2.5)^*^	92.5(2.5)	90.5(3)^**^
mpip			66(1.5)	64.5(1.5)^**^
rpip	68(1.5)	67(1.5)^*^	65(1.5)	64(2)^**^

	normalized movement time (%)							
	40		50		60		100	
	ol	sl	ol	sl	ol	sl	ol	sl
joint								
imcp	−4(4)	−2.5(3)^*^	−13(3.5)	−11.5(3.5)^*^	−17(3.5)	−16(3.5)^*^	−2(3.5)	−1(3.5)^*^
mmcp	2(2)	3.5(2)^*^	−3.5(2)	−2(2)^**^	−6(2)	−5(2)^*^		
rmcp			−9.5(2)	−8.5(2)^**^				
tpip			0(1)	1(1)^**^				
ipip	65(3)	66.5(3)^*^						

Mean standard errors are reported in parentheses. The relevant statistical comparisons are between the ‘OL’ and the ‘LL’ conditions and between the ‘OL’ and the ‘SL’ conditions.

**Notes.** Only significant results are reported (* = *p<*.05; ** = *p<*.01). OL = Odourless air - Large target; LL = ‘Large’ odour - Large target; SL = ‘Small’ odour - Large target. Tmcp = metacarpal joint of the thumb; Imcp = metacarpal joint of the index finger; Mmcp = metacarpal joint of the middle finger; Rmcp = metacarpal joint of the ring finger; Tpip = proximal interphalangeal joint of the thumb, Ipip = proximal interphalangeal joint of the index finger; Rpip = proximal interphalangeal joint of the ring finger.

**Figure 6:**
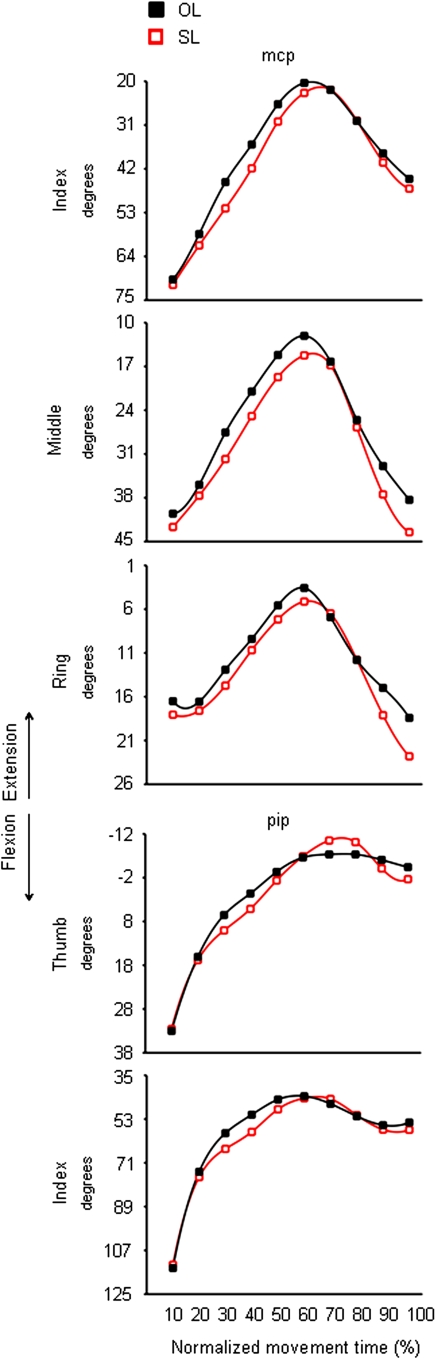
Time course of finger motion during reaching for the large target either in the absence or in the presence of an odour evoking a small object. Each trace denotes the average angular excursion of a representative subject (subject 15) for the *mcp* joint of index, middle and ring fingers (upper panels), and the *pip* joint of the thumb and index finger (lower panels) when performing the ‘OL’ (black squares) and the ‘SL’ (red squares) conditions. Positive values correspond to finger flexion whereas negative values correspond to finger extension. OL = Odourless air-Large target; SL = ‘Small’ odour-Large target.

**Figure 7:**
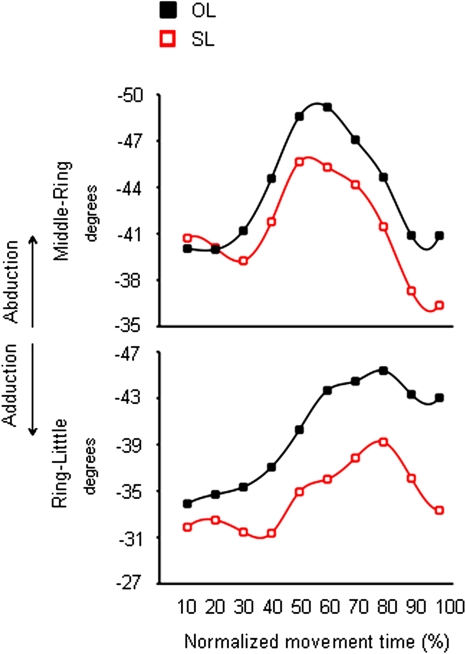
Time course of abduction angle between fingers during reaching for the large target either in the absence or in the presence of an odour evoking a small object. Each trace denotes average abduction angle of a representative subject (Subject 10) for the middle-ring and the ring-little fingers when performing the ‘OL’ (black squares) and the ‘SL’ (red squares) conditions. Increase in negative values correspond to bigger abduction (i.e., increase of digits' angular distance). OL = Odourless air-Large target; SL = ‘Small’ odour-Large target.

For the incongruent ‘SL’ condition, the *mcp* joint of the index, middle, and ring fingers was more flexed than for the ‘OL’ condition (Fig. 6). In particular, the *mcp* joint of index, middle, and ring fingers showed an over-flexion at about half of movement duration (Table 1). However, a delayed odour ‘size’ effect was evident for the *mcp* joint of the index finger (Table 1). A similar pattern was also found for the *pip* joints of both the thumb and the index finger showing a greater flexion in the ‘SL’ than in the ‘OL’ condition at 50% and 40% of movement duration, respectively (Fig. 6 and Table 1). The middle-ring and the ring-little abduction angles were smaller for the ‘SL’ than for the ‘OL’ condition. This effect was evident within the second half of movement duration (Fig. 7 and Table 2).

**Table 2:** Average fingers' abduction angles at different epochs of the normalized movement time.

	normalized movement time (%)											
	50		60		70		80		90		100	
	ol	sl	ol	sl	ol	sl	ol	sl	ol	sl	ol	sl
abduction angle												
middle-ring	−31(1.5)	−30(1.5)	−31.5(2)	−30.5(1.5)	−31.5(2)	−30.5(1.5)			−29.5(2.5)	−28.5(2)	−29(2.5)	−27.5(2.5)
ring-little							−30(2.5)	−29(2.5)				

Mean standard errors are reported in parentheses.

The relevant statistical comparisons are between the ‘OL’ and the ‘SL’ conditions.

**Notes.** Only significant results are reported (*p<*.05). OL = Odourless air- Large target; SL = ‘Small’ odour-Large target.

These results indicate that the presence of a ‘large’ odour magnified the ‘extension’ pattern which was found when a large target was grasped in the absence of olfactory information. Such magnification was particularly evident during the first part of movement duration. Conversely, the presence of a ‘small’ odour determined a ‘flexion’ pattern which was not evident when the large target was grasped in the absence of olfactory information (showing a similarity, in terms of flexion, with the pattern elicited by the small target when grasped in the absence of olfactory information). The effect due to the presence of the ‘small’ odour persisted up to the end of the movement duration.

#### Grasping a small target

**Figure 8:**
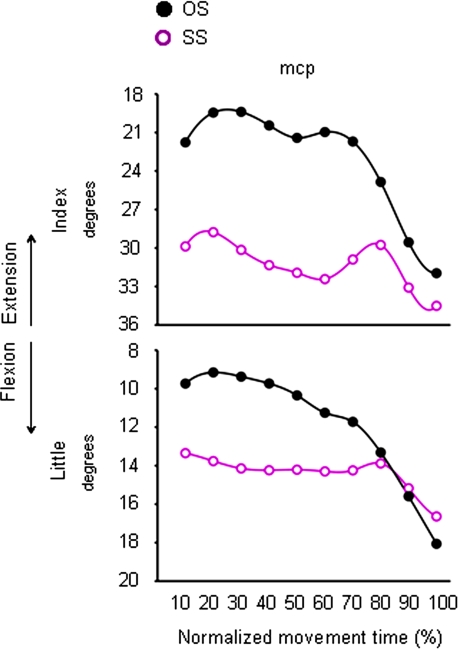
Time course of finger motion during reaching for the small target either in the absence or in the presence of an odour evoking a small object. Each trace denotes average angular excursion of a representative subject (Subject 2) for the *mcp* joint of the index and the little fingers when performing the ‘OS’ (black circles) and the ‘SS’ (purple circles) conditions. Positive values correspond to finger flexion whereas negative values correspond to finger extension. OS = Odourless air-Small target; SS = ‘Small’ odour-Small target.

**Figure 9:**
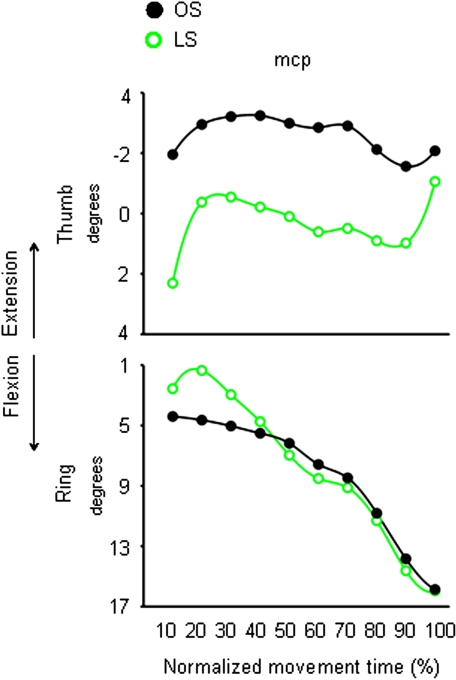
Time course of finger motion during reaching for the small target either in the absence or in the presence of an odour evoking a large object. Each trace depicts average angular excursion of a representative subject (Subject 2) for the *mcp* joint of the thumb and the ring finger when performing the ‘OS’ (black circles) and the ‘LS’ (green circles) conditions. Positive values correspond to finger flexion whereas negative values correspond to finger extension. OS = Odourless air-Small target; LS = ‘Large’ odour-Small target.

For the congruent ‘SS’ condition, the *mcp* joints of both the index and the little finger were more flexed than for the ‘OS’ condition. Specifically, the *mcp* joints for both the index and the little finger showed such over-flexion at 40%, and from 20 up to 60% of movement duration, respectively (Fig. 8 and Table 3). For the incongruent ‘LS’ condition, angular excursion of the *mcp* joint for both the thumb and the ring finger significantly differed from angular excursions obtained for the ‘OS’ condition. In particular, at 20% of movement duration, the *mcp* joint of the ring finger was more extended in the ‘LS’ than ‘OS’ condition (Fig. 9 and Table 3). In contrast, from 10% up to the end of movement duration, the *mcp* joint of the thumb was more flexed for the ‘LS’ than for the ‘OS’ condition (Fig. 9 and Table 3).

**Table 3:** Average angular excursions at different epochs of normalized movement time.

	normalized movement time (%)							
	20		40		50		60	
	os	ss	os	ss	os	ss	os	ss
joint								
Imcp			14(3.5)	16(3.5)^*^				
Lmcp	−5(2)	−4(2)^**^	−6(2)	−5(2)^**^	−9(2.5)	−8(2.5)^***^	−12.5(3)	−11.5(3)^**^

	normalized movement time (%)															
	10		20		40		50		60		80		90		100	
	os	ls	os	ls	os	ls	os	ls	os	ls	os	ls	os	ls	os	ls
joint																
tmcp	−6.5 (2)	−5.5(2)^**^			−10.5(2)	−9.5(2)^*^	−12(2)	−11(2)^***^	−13.5(2)	−12.5(2)^***^	−14(2)	−13(2)^*^	−13.5(2)	−12.5(2)^*^	−13(2)	−12(2)^**^
rmcp			−1.5(2)	−2.5(2)^*^												

Mean standard errors are reported in parentheses. The relevant statistical comparisons are between the ‘OS’ and the ‘SS’ conditions and the ‘OS’ and the ‘LS’ conditions.

**Notes.** Only significant results are reported (* = *p*<.05; ** = *p*<.01, *** = *p*<.001). OS = Odourless air - Small target; SS = ‘Small’ odour - Small target; LS = ‘Large’ odour - Large target. Tmcp = metacarpal joint of the thumb; Imcp = metacarpal joint of the index finger; Rmcp = metacarpal joint of the ring finger; Lmcp = metacarpal joint of the little finger.

To sum up, the presence of a ‘small’ odour enhanced the pattern of hand flexion which was found when the small target was grasped in the absence of olfactory information. Such intensification was particularly evident during the first part of movement duration. Conversely, the presence of a ‘large’ odour determined both a greater ring finger extension and a greater thumb flexion with respect to when the small target was grasped in the absence of olfactory information (showing similarity with the pattern elicited by the large target when grasped in the absence of olfactory information). The effect due to the presence of the ‘large’ odour persisted throughout the entire movement duration.

### Hand Motion Covariation

This section reports on the results concerned with the pattern of hand motion covariation as obtained by the absolute value of the slopes of the regression lines fitting angular values between articulations' pairs (see ‘Data analysis’ section). The relationship between the size of the odour-evoked stimulus and the size of the visual target did affect the absolute value of the slopes during reaching (F_(6.36,572.25)_ = 4.02, *p*<.001). Post-hoc analyses revealed that the slope absolute values decreased at specific epochs during reaching only when the odour was associated with an object having a different size than the visual target (Table 4). Further, the temporal window of the reduction in covariation was wider when the stimulus associated with the odour was small rather than large (Table 4). Therefore, the pattern of hand motion covariation was weakened when the ‘size’ of the odour did not match the size of the target. Importantly the delivery of an odour evoking a stimulus of a similar size to the target did not alter the motion covariation characterizing the hand when no odour was delivered.

**Table 4:** Average absolute value of the slopes of the regression lines fitting angular values for each articulations' pair at different epochs of normalized movement time.

normalized movement time (%)							
40		50		60		70	
ol	sl	ol	sl	ol	sl	ol	sl
0.33(0.02)	0.30(0.02)	0.33(0.02)	0.29(0.02)	0.31(0.02)	0.28(0.02)	0.30(0.02)	0.28(0.02)

normalized movement time (%)			
20		40	
os	ls	os	ls
0.34(0.03)	0.33(0.03)	0.34(0.03)	0.30(0.03)

Mean standard errors are reported in parentheses.

**Notes.** Only significant results are reported (*p<*.01, *FDR* correction). OL = Odourless air- Large target; SL = ‘Small’ odour -Large target; OS = Odourless air-Small target; LS = ‘Large’ odour-Large target.

## Discussion

The present study has investigated the effects of odour stimuli on the kinematics of hand shaping at the level of individual digits' motion. The results indicate that the kinematic patterning of a reach-to-grasp movement was influenced by the ‘size’ of an odour. Crucially, the motor plan evoked by the odour is surprisingly fine-grained and when elicited can modulate both the pattern of angular excursion at the level of individual fingers' joints and the degree of synergic movement amongst digits.

### When the Size of the Visual Target and the ‘Size’ of the Olfactory Stimulus do not Match Interference Emerges

As reported here, reach-to-grasp movements can be planned on the basis of olfactory information. The motor plan elicited by the olfactory stimulus is not totally overridden by the motor plan triggered, at a later time, by the visual target. That is, some aspects of the motor plan elicited by a ‘size’ incongruent olfactory stimulus persist in the motor plan executed for grasping the visual target. This effect was evident when comparing the incongruent odour (‘LS’ and ‘SL’) with the respective odourless (‘OS’ and ‘OL’) conditions.

When the odour was ‘large’ and the visual target was small, only one finger joint (i.e., the *mcp* joint of the ring finger) was affected by the olfactory stimulus. In contrast, the influence of the ‘small’ odour on the kinematics of a reach-to-grasp movement towards a large target was much more evident and a greater number of joints were mobilized. This seems to suggest that planning for a reach-to-grasp movement on the basis of a ‘small’ odour when the target is large poses more constraints than when the odour is ‘large’ and the movement is directed towards a small target. Our proposal is that the motor plan elicited by the odour has to be modified according to the visual target. However such reorganization could be more easily managed without compromising object grasp when the odour is ‘large’ and the visual target is small than vice versa.

In terms of complexity, several factors could contribute to the difference in kinematic response between the two types of incongruent conditions. For instance, biomechanically there may be more advantage for closure (as happens for the present ‘LS’ condition) than for opening (as happens for the present ‘SL’ condition). Colebatch and Gandevia [28] found, for example, that thumb and finger flexors were 2.8–3.5 times stronger than extensors. For a task focused upon a grasping action, the biomechanical setting for the flexors would be more favoured. This view seems to be supported by the results obtained in previous studies looking at the reprogramming of grip aperture following a perturbation of object size [29], [30]. These findings indicate that the passage from a large to a small object was easier than the passage from a small to a large object.

Of note is the finding that when the odour is ‘large’ and the target is small, the thumb is over-flexed with respect to the condition in which the small visual stimulus is presented without preceding olfactory information. A possible explanation for such an effect considers how the thumb behaves for movements performed in the absence of olfactory information (i.e., no-odour conditions). In such circumstances, the thumb is usually more flexed at the end of the movement for the large than for the small target. Therefore, the finding that a ‘large’ odour determines an over-flexion of this digit strengthens the hypothesis that a motor plan suited for grasping a larger target is evoked by the odour.

The delivery of ‘incongruent’ odours also had an effect on the extent of synergic movements within the hand. This is signified by the loosening of synergies amongst fingers observed for the incongruent odour conditions with respect to the level of synergies observed for the no-odour conditions. A possible interpretation for these findings relies on the requirement to integrate the motor plan established for the visual target into the motor plan elicited by the preceding ‘odour’ stimulus. This integration process is gradual and it spreads throughout the entire movement duration. In other words, the ‘olfactory’ motor plan is not immediately excluded as the visual target appears (as it can be noticed on the fingers' angular excursion profiles), but penetrates the ‘visual’ motor plan. Such intrusion results in an on-line adjustment which renders the system more unstable and therefore determines a decrease in the level of covariation amongst digits. In line with the hypothesis that dealing with the intrusion of a ‘large’ odour is easier than dealing with the intrusion of a ‘small’ odour, the temporal window in which the decrease in the level of covariation was found it was greater when the olfactory stimulus was ‘small’ and the visual target was ‘large’ than when the olfactory stimulus was ‘large’ and the visual target was ‘small’.

It is now necessary to comment on how we view the processing of olfactory stimuli in terms of action control. Our preferred ideas are that during initial perceptual analysis, a limited number of objects potentially relevant for action are processed in parallel. This initial perceptual processing flows continuously into areas of the brain that represent and subsequently initiate action. Such perceptual inputs are capable of automatically activating their associated responses without subjects' intentions to act [31], [32]. Due to this highly efficient and automatic conversion of perceptual inputs into the actions, different sensory inputs can evoke actions in parallel. As soon as the target is identified, an appropriate reach-to-grasp motor plan is initialized which then competes with the motor plan triggered by the odour; this conflict is played out in the kinematics of hand shaping. Thus, according to this model, the difference between the grasp plans activated by the visual target and by the olfactory stimulus is essential for hand shaping interference effects to be observed.

### When the Size of the Visual Target and the ‘Size’ of the Olfactory Stimulus Match Facilitation Emerges

When a preceding odour elicits a motor plan which is congruent with the motor plan subsequently established for the visual target, the kinematic patterning is magnified. Therefore, the grasp plan triggered by the olfactory stimulus primed the grasp plan established for the visual target. This effect was evident at the very beginning of the movement, fading away during the second phase of the movement. Remember that for both the incongruent conditions the conflict between the ‘olfactory’ and the ‘visual’ grasp plans lasted for the entire movement duration. Importantly, and again in contrast with what reported for the incongruent conditions, an odour of a similar ‘size’ than the visual target, does not alter hand synergies with respect to when no-odour is presented. This indicates that when the ‘size’ of the odour and the size of the visual target match, the integration of the two modalities reinforces the grasp plan, the established synergic pattern is more ‘protected’ and it does not change. Having two sources carrying similar information leads to a more stable and coherent action.

Research on multisensory processing brings evidence of enhancements of multimodal neurons' firings, perceptual processes, or reaction times, in response to stimuli with similar characteristics represented in different modalities [1], [33]–[36]. More recently, similar enhancements have also been found for prehensile tasks [16]–[18]. For instance, reach-to-grasp movements were faster if two cues related to the same target object pertained to different sensory modalities, i.e., visual and auditory than when only one cue is presented [18]. The present results crucially extend this literature by demonstrating that similar facilitation effect can also be revealed for multisensory integrations involving olfaction.

It is tempting to speculate about the possible neural mechanisms underlying the reported facilitation effects. Evidence from neuroimaging [37], [38] and neurophysiological studies [39]–[41] may help in this exercise. For example, by manipulating the degree of semantic correspondence between odour-picture pairs, Gottfried and Dolan [37] revealed facilitation for semantically congruent versus incongruent situation. This advantage was associated with enhanced neural activity within the orbitofrontal cortex (OFC). Similarly, Österbauer and collaborators [38] found increased activity within the OFC when the perceived congruence between visual and olfactory stimuli became progressively higher. Thus, it might be reasonable to assume that the facilitation effects found in the present study are mediated by visual-olfactory representations encoded at the level of multisensory integration sites within the OFC. But, how do these visual-olfactory representations manage to modulate motor output? Comparative literature may provide some evidence for neural networks which connect the OFC with motor regions [42]. Of particular interest for our study is the presence of direct connections between OFC and motor areas involved in arm-hand movement control such as the motor cingulated area 24c/M3, the supplementary motor area F3/M2, the pre-supplementary motor area F6 and the ventral premotor area F5. Furthermore, also the primary motor cortex (M1) receives inputs from frontal granular area 12 [43]. On the basis of the well-known homology between cerebral regions underling reach-to-grasp movement in monkeys and humans [8], [44], we suggest that the cortico-cortical connections between OFC and motor areas influencing motor output in non human primates [45] may also exist in humans and account for the influence of multisensory information on motor behaviour and more specifically on prehensile actions [46]. In this respect, the present findings provide some support to theoretical models specifically designed to infer about the neural mechanisms underlying reach-to-grasp movements [47], [48]. These models posit that robust ‘multisensory’ perception might act to increase the level of activation of perceptual schemas, which in turn might increase the ‘readiness‘ of brain areas devoted to the control of prehensile actions. In this view, we demonstrate that also olfactory information, as with any sensory modality, might have the potential to enhance activity within the neural networks subtending a complex system such as the hand.

### Conclusions

A tenet from previous research on reach-to-grasp movements is the notion of visuo-motor transformation. That is, the conversion of the geometric features characterizing the to-be-grasped object into an appropriate motor prototype. The evidence for the existence of such process comes from the demonstration that structural properties (e.g., size, shape, and texture) of visually encoded objects reflect on hand posture at the level of individual finger movements when grasping.

Here we extend this notion revealing that the size of the object evoked by the odour has the potential to modulate hand shaping. Importantly, the fact that ‘size’ olfactory information modulates the hand at the level of individual digits (and not only the thumb-index distance as previously reported) leads to two important considerations in terms of sensorimotor transformation. First, from a perceptual perspective, the representation evoked by the odour seems to contain highly detailed information about the object (i.e., volumetric features rather than a linear dimension such as the thumb-index distance). If olfaction had provided a blurred and holistic object's representation (i.e., a low spatial-resolution of the object's image), then the odour would have not affected the hand in its entirety. Second, from a motor perspective, the olfactory representation seems to be mapped into the action vocabulary with a certain degree of reliability. The elicited motor plan embodies specific and selective commands for handling the ‘smelled’ object, and it is fully manageable by the motor system. Therefore, it is not an incomplete primal sketch which only provides a preliminary descriptive in the terms of motor execution.

Another aspect of the present results is how hand kinematics modulates depending on the similarity between the ‘visual’ and ‘olfactory’ motor blueprints. Current literature on multisensory integration reports facilitation effects when two sensory modalities provide congruent information about an object and interference effects when different sensory modalities provide discordant information. In this respect, we crucially extend this literature by having identified a chemosensory-visual binding for the control of action. We found facilitation effects when olfactory/visual information elicited a congruent motor planning and interference when olfactory/visual information triggered different motor plans.

The present findings open to a number of unsolved questions. For instance, how do multisensory integration neural loci, such as the orbitofrontal cortex, modulate their activity when information for action planning is provided through different modalities? And, how do multisensory integration sites ‘talk’ with the neural circuits underlying grasping as to modulate motor output? Further research using functional imaging and neurophysiological techniques may have the potential to uncover the neural underpinnings for the effects reported here.

## Materials and Methods

### Subjects

Twenty-six right handed subjects (21 females and 5 males, mean age 22±3.5 years) took part in the experiment. All participants reported normal olfaction, no history of olfactory dysfunction, and normal or corrected-to-normal vision in a confidential report. All subjects were naïve as to the purpose of the experiment and gave their informed written consent to participate in the study. The experimental session lasted approximately 30 min. The experimental procedures were approved by the Institutional Review Board at the University of Padua and were in accordance with the declaration of Helsinki.

### Stimuli and apparatus

The visual stimuli (i.e., targets) consisted of four plastic objects grouped on the basis of their natural size: large (apple, orange) and small (almond, strawberry) (Fig. 1A). Plastic objects were used in order to maintain consistent visual attributes and sizes similar throughout the period of experimentation. The odour stimuli corresponded to the target stimuli described above. Odour solutions of strawberry, almond, orange, and apple were obtained mixing 6000 µl of prophylenic glycol and 180 µl (3%), 60 µl (1%), 420 µl (7%), and 45 µl (0.75%) of the specific odorant compound, respectively. A custom-built computer-controlled olfactometer (Department of Experimental Psychology, University of Oxford) was used to deliver the odour stimuli or odourless air. Each odour generator consisted of a glass boat containing one of the four odour stimuli. A fifth glass boat containing prophylenic glycol was used for the delivery of odourless air. The air passed over the odour solutions and the prophylenic glycol at a flow rate of 8 l/min and it was delivered to subjects via Teflon tubing to a facial mask (Fig. 1B). Data from a pilot study showed that the objects associated with the administered odour stimuli were all correctly identified by the subjects. Further, the odour stimuli were judged to have equal intensity, hedonic tone and familiarity and to be iso-intense during all the experimental session. At the beginning of each trial, subjects placed their right hand on a starting platform within which a pressure sensitive switch was embedded (i.e., starting switch). The platform was designed with slight convexities dictating a natural flexed posture of the fingers (Fig. 1B). The target object was placed on a second pressure sensitive switch (i.e., the ending switch) embedded within the working surface (Fig. 1B).Vision was controlled using spectacles fitted with liquid crystal lenses (Translucent Technologies Inc., Toronto, Ontario, Canada) that rendered the target visually accessible by changing from opaque to clear (Fig. 1B). The release of the starting switch corresponded to the onset of the reaching movement towards the target and determined visual availability of the target object (i.e., opening of the spectacles). Movement offset was taken at the time in which the ending switch was released when the object was lifted. Reaching duration was calculated as the time interval between the release of the starting and ending switches.

### Procedures

Participants began each trial with the elbow and the wrist resting on a flat surface, the forearm horizontal, the arm oriented in the parasagittal plane passing through the shoulder, and the right hand in a pronated position with the palm toward the working surface on the starting switch. The target was aligned with the subject's body midline and located at 33-cm-distance from the hand starting position to the left of the subject's right shoulder (Fig. 1B). The sequence of events for each trial was as follows: 1) vision was occluded before the target was positioned on the working surface; 2) an auditory tone (850 ms duration, 65 dB sound pressure, and 800 Hz frequency) indicated odour delivery; 3) after 3 s, a similar tone indicated the offset of odour delivery; 4) following a 500 ms interval the tone was presented again; 5) upon hearing the tone, participants were instructed to reach towards, grasp and lift the target object. Sufficient time (10 s) was allowed between trials to recover from any odour adaptation [49]. The adopted sequence of events was chosen because previous literature has revealed that effects of task irrelevant information on reach-to-grasp kinematics are maximized when the task irrelevant stimulus/cue (presented in the same or a different sensory modality than the target) is presented slightly before the to-be-grasped target [16], [31], [50]. We instructed the subjects to reach at a natural speed and not to grasp the object by the stem. The experimenter visually monitored each trial to ensure subject's compliance to these requirements. Subjects naturally grasp the small objects between the thumb and either (or both) the index and the middle fingers and the large objects opposing the thumb with all the other fingers.

This experimental task was performed under six different experimental conditions:

(1) ‘OL’ condition: odourless air was delivered before the reach-to-grasp movement towards a large target was initiated;

(2) ‘OS’ condition: odourless air was delivered before the reach-to-grasp movement towards a small target was initiated;

(3) ‘LL’ condition: an odour associated with an object of a large size was presented before the reach-to-grasp movement towards a large target was initiated;

(4) ‘SS’ condition: an odour associated with an object of a small size was presented before the reach-to-grasp movement towards a small target was initiated;

(5) ‘SL’ condition: an odour associated with an object of a small size was presented before the reach-to-grasp movement towards a large target was initiated;

(6) ‘LS’ condition: an odour associated with an object of a large size was presented before the reach-to-grasp movement towards a small target was initiated.

Odour-target combinations for each experimental condition are represented in Fig. 2. Participants performed a total of 48 trials (8 for each experimental condition) which were presented in randomized order within one block.

### Recording techniques

Hand posture was measured by resistive sensors embedded in a glove (CyberGlove, Virtual Technologies, Palo Alto, CA, USA), worn on the subject's right hand (Fig. 1B). The sensors' linearity was 0.62% of maximum nonlinearity over the full range of hand motion. The sensors' resolution was 0.5°, which remains constant over the entire range of joint motion. The output of the transducers was sampled at 12-ms interval. Angular excursion was measured at metacarpal phalangeal (*mcp*) and proximal interphalangeal (*pip*) joints of the thumb, index, middle, ring, and little finger. Abduction angles between the thumb-index, index-middle, middle-ring, and ring-little fingers were measured. Before the experimental block started, baseline hand posture for each subject was recorded. Subjects were requested to place their right hand flat on the table with the fingers straightened, close to each other and to hold that position until baseline fingers' angular excursion and abduction angles were recorded. Angular excursion and abduction angles were defined 0° when the fingers were maintained straight and together in the plane of the palm (‘reference hand posture’). Fingers' flexion was assigned positive values whereas fingers' extension was given negative values with respect to the baseline. Abduction angles were reported on a continuum of negative values with respect to the baseline. A decrease in such values indicated relatively greater abduction.

### Data Analysis

Data from each trial were time normalized to compare hand posture across experimental conditions at different epochs during reaching. Specifically, the pattern for both fingers' angular excursion and abduction angles was calculated from 10 to 100% of reaching duration, at 10% intervals. The results predicted by our hypotheses (see ‘Introduction’ section) were assessed at each epoch of the normalized movement time by means of planned orthogonal contrasts [51] implemented with R-2.5.1 software package (http://cran.r-project.org). Since contrasts are coding vectors that mathematically express predicted results [52], we created vectors to assess the target size effect (i.e., 1 and −1 for ‘OL’ and ‘OS’ condition, respectively, 0 for the remaining conditions), the effect of odours having a similar ‘size’ as the visual target (i.e., 1 and −1 for ‘LL’ and ‘OL’ condition, respectively, 0 for remaining conditions; −1 and 1 for ‘SS’ and ‘OS’ condition, respectively, 0 for remaining conditions), and the effect of odours having a different ‘size’ than the visual target (i.e., −1 and 1 for ‘SL’ and ‘OL’ condition, respectively, 0 for remaining conditions; 1 and −1 for ‘LS’ and ‘OS’ condition, respectively, 0 for remaining conditions). We used one-tail *t-test* for all fingers' joints and abduction angles since a specific direction of the ‘size’ effect for both the target and the object evoked by the odour was predicted. A two-tails *t-test* was used for the thumb's joints given that on the basis of recent experimental evidence no specific predictions could be made [53], [54]. This is because it has been demonstrated that the thumb's angular excursion is not specifically modulated to object's structural properties (e.g., shape), but it reflects a role in action guidance. The *t-*values corresponding to each contrast were considered statistically significant if less than .05 (α-level).

The effects of the relationship between the ‘odour’ size and the visual target size on the degree of motion covariation within the hand during reaching for the target were assessed as follows. First, we computed the slope of the regression line between angular excursion of ‘joint-joint’, ‘joint-abduction’, and ‘abduction-abduction’ pairs (45, 40, and 6 pairs, respectively, for a total of 91 pairs) for each of the six experimental conditions (i.e., ‘OL’, ‘OS’, ‘LL’, ‘SS’, ‘SL’, ‘SS’) and for each epoch of the normalized movement time. For this analysis, each subject was taken as a statistical unit. Then, in order to obtain a quantitative index of the degree of hand motion covariation, we calculated absolute values of the obtained slopes. Finally, an analysis of variance (ANOVA) was performed on these values with odour ‘size’ (large, small, and no-odour), target size (large, small), and time (from 10 to 100%, by step of 10%) as within subject factors. For this analysis, each of the 91 pairs was considered as a statistical unit. Before running the ANOVA, we checked for all the main assumptions behind this statistical model (i.e., normality and sphericity). Kolmogorov-Smirnov test revealed that the normality assumption was satisfied (α-level: .05). Whereas, Mauchly test showed that the sphericity assumption was violated (α-level: .05), hence, Greenhouse-Geisser correction was applied to the degrees of freedom of *F*-statistics.

Results from the ANOVA performed on the slope absolute values were explored through post-hoc multiple comparisons. Specifically, paired sample *t-tests* were used to compare ‘OL’ vs. ‘OS’ condition, ‘LL’ vs. ‘OL’ condition, ‘SL’ vs. ‘OL’ condition, ‘SS’ vs. ‘OS’ condition, and ‘LS’ vs. ‘OS’ condition at each epoch. For these *t-tests*, the increase of the type I error (α-level: .01) was controlled by applying False Discovery Rate (*FDR*) correction [55].
